# An Exceptionally Complex Chromosome Rearrangement in the Great Tit *(Parus major)*: Genetic Composition, Meiotic Behavior and Population Frequency

**DOI:** 10.3390/cells15010052

**Published:** 2025-12-27

**Authors:** Anna Torgasheva, Lyubov Malinovskaya, Miroslav Nuriddinov, Kira S. Zadesenets, Maria Gridina, Artem Nurislamov, Svetlana Korableva, Inna Pristyazhnyuk, Anastasiya Proskuryakova, Katerina V. Tishakova, Nikolay B. Rubtsov, Veniamin S. Fishman, Pavel Borodin

**Affiliations:** 1 Institute of Cytology and Genetics, Russian Academy of Sciences, 630090 Novosibirsk, Russia; l.malinovskaia@g.nsu.ru (L.M.); numiabnew@gmail.com (M.N.); kira_z@bionet.nsc.ru (K.S.Z.); gridinam@gmail.com (M.G.); a.nurislamov@alumni.nsu.ru (A.N.); s.kozyreva@g.nsu.ru (S.K.); iprist@bionet.nsc.ru (I.P.); tishakova@mcb.nsc.ru (K.V.T.); rubt@bionet.nsc.ru (N.B.R.); minja-f@yandex.com (V.S.F.); borodin@bionet.nsc.ru (P.B.); 2 Centre for Molecular Biodiversity Research, Leibniz Institute for the Analysis of Biodiversity Change, Museum Koenig Bonn, 53113 Bonn, Germany; 3 Bonn Institute for Organismic Biology-Animal Biodiversity, University of Bonn, 53121 Bonn, Germany; 4 Laboratory of Genome Structure and Function, Novosibirsk State University, 630090 Novosibirsk, Russia; 5 Genetics and Life Sciences, Sirius University of Science and Technology, Sirius Federal Territory, 354340 Sochi, Russia; 6 Institute of Molecular and Cellular Biology, Russian Academy of Sciences, 630090 Novosibirsk, Russia; andrena@mcb.nsc.ru

**Keywords:** structural variation, chromosomal inversions, copy number variation (CNV), great tit, genome evolution, recombination suppression, FAM118A, Hi-C

## Abstract

**Highlights:**

**What are the main findings?**

- A complex rearrangement on chromosome 1A, combining a ~55 Mb inversion and >30 Mb of copy-number expansion, is present in ~19% of the great tits in the Siberian population. A similar rearrangement has been previously detected in 5% of birds in the European population.
- The amplified region includes a sequence homologous to the *FAM118A* locus conserved in vertebrates with a nested 630 bp tandem repeat, expanded to ~50,000 copies in the rearranged chromosome.

**What are the implications of the main findings?**

- The rearranged chromosome provides a natural system to investigate the genomic consequences and evolutionary maintenance of large inversion–amplification complexes.
- The *FAM118A* expansion provides a framework for assessing potential functional and adaptive effects of extreme gene amplification in a wild population.

**Abstract:**

Chromosomal inversions and copy-number variants (CNVs) drive genomic and phenotypic diversification in birds by reshaping recombination, gene expression, and genome architecture. Here, we report a complex structural polymorphism on great tit (*Parus major*) chromosome 1A that occurs in the Siberian population with a 19% heterozygote frequency. Using cytogenetic and genomic approaches, we show that this rearrangement combines a ~55 Mb paracentric inversion in the long arm with a dramatic (>30 Mb) expansion of the short arm driven by extensive amplification of multiple genomic loci. These include a region homologous to the poorly characterized *FAM118A* gene, whose paralog *FAM118B* has been recently shown to play a pivotal role in innate immune activation. This region is missing from the current reference genome assembly while present in ~20 copies on wild-type 1A chromosome and nearly twentyfold amplified in the rearranged variant. It contains a nested 630 bp tandem repeat, encompassing the entire exon 3, which has burst to a total of ~50,000 copies in the rearranged chromosome. While functional analyses are required to uncover the biological effects of the genomic features linked to this rearrangement, our results offer a unique framework for studying how complex structural polymorphisms drive genome innovation and adaptive diversity.

## 1. Introduction

Chromosomal rearrangements, including inversions, translocations, and copy-number variations (CNVs), play an important role in genome evolution by altering recombination patterns, gene expression, and genomic stability. Among these, inversions are particularly important because they suppress recombination in heterozygotes, thereby maintaining linkage between co-adapted alleles and facilitating local adaptation [1,2,3]. In many taxa, inversions have been linked to ecologically relevant traits, reproductive isolation, and speciation [4]. On the other hand, the suppression of recombination in the heterozygotes is usually accompanied by the accumulation of deleterious mutations (Muller’s ratchet). Delayed synapsis of the inverted and adjacent regions and retarded repair of the DNA double-strand breaks (DSBs) also contribute to the increased mutational load in the rearranged chromosomes [5].

Despite their potential deleterious effect, inversions are the most common chromosome rearrangements detected in otherwise conservative karyotypes of birds [6,7]. Cytological and molecular genetic analyses revealed intraspecies polymorphism and interspecies differences for inversions. Meta-analysis of the karyotypes of 411 passerine species demonstrated a substantial contribution of inversions in speciation [8]. Two textbook examples of adaptive inversion polymorphism in birds were described in white-throated sparrows (*Zonotrichia albicollis*) [9] and ruffs (*Calidris pugnax*) [10]. Inversion polymorphism in zebra finch (*Taeniopygia guttata*) and willow warbler (*Phylloscopus trochilus*) has been ascribed to balanced and divergent selection [11,12]. Recent studies in the zebra finch demonstrated that many balanced inversion polymorphisms have been maintained at intermediate frequencies for a long time, most of them being structurally complex and often containing smaller nested or overlapping inversions [13].

Inversion breakpoints are often saturated with repetitive DNA elements and segmental duplications for two reasons [14,15,16]. On the one hand, dispersed repeated sequences facilitate the occurrence of inversions via ectopic recombination. On the other hand, inversions themselves facilitate structural variation around their breakpoints through non-allelic homologous recombination [17,18]. Recombination between inverted segmental duplications can mediate inversions by flipping the intervening sequence, whereas unequal recombination between direct duplications leads to deletions or duplications [19]. This dynamic process, sometimes referred to as “inversion toggling,” destabilizes the region by interfering with proper homologous pairing and promoting the formation of copy-number variants (CNVs) [20,21,22]. As a result, inversions and CNVs frequently co-occur, forming complex rearrangements that may profoundly alter genome architecture [23,24].

Beyond their structural impact, CNVs may have direct phenotypic and adaptive consequences when encompassing gene-containing regions. Such rearrangements play a significant role in adaptation and speciation by providing a source for genetic variation that can rapidly respond to selective pressures [25]. Duplications of existing genes can lead to the creation of novel gene copies, potentially diversifying gene repertoires and leading to new traits that are advantageous in a changing environment [26]. They can alter gene expression or inhibit gene function, driving adaptive divergence contributing to local adaptations and reproductive isolation [27,28,29].

Despite their generally compact genomes, birds exhibit a surprisingly high prevalence of CNVs, which have been linked to immune responses, sensory adaptations, and life-history traits [30,31]. They play a key role in immune system evolution, particularly in the diversification of the major histocompatibility complex (MHC). The ancestral state had low MHC copy numbers, but passerines evolved higher copy numbers through gene duplications, likely as adaptation to diverse pathogens [32]. CNVs affecting MHC genes have also been linked to lifespan, migratory behavior, and ecological specialization, underscoring their potential importance for avian adaptation [33,34].

The great tit (*Parus major*) is one of the best-studied passerine species for evolutionary and ecological genomics [35]. With a wide Eurasian distribution and extensive phenotypic variation, it has served as a model for investigating the genetic basis of behavior, reproduction, and adaptation to climatic gradients [36,37,38]. The availability of a reference genome [35], large-scale resequencing datasets [39], and detailed ecological monitoring across multiple populations makes this species an ideal system for exploring the role of structural variation in natural populations.

A large-scale structural polymorphism was recently described in European populations of the great tit. Using population-genomic data, da Silva et al. [40] identified a large inversion on chromosome 1A encompassing nearly the entire chromosome length. The rearranged variant occurred at low frequency (~5%) and was associated with a complex copy-number variation (CNVR 2802) near the inversion breakpoint. However, the structure of this rearrangement has not been confirmed cytogenetically.

During routine karyotyping of great tits from a Siberian population, we serendipitously discovered several heterozygotes for a complex rearrangement in chromosome 1A that combines an inversion with extensive amplification of genetic material. To comprehensively characterize this rearrangement, we applied a combination of cytogenetic and genomic approaches. We examined pairing patterns and recombination distribution in female and male heterozygotes using immunolocalization of key meiotic proteins. We delineated the inversion borders using Hi-C contact maps and identified the source of the massive amplification using whole-genome sequencing with short and long reads. A prominent component of the amplification was a sequence homologous to *FAM118A*, a poorly characterized but highly conserved gene missing from the great tit reference genome assembly. We characterized the structure of this locus and the degree of its amplification in the wild-type and rearranged chromosomes. The results allowed us to conclude that this rearrangement represents the same PMA1A variant previously described in European populations [40], though present at about three times higher frequency in the Siberian population.

## 2. Materials and Methods

### 2.1. Animals

A total of 46 great tits were used in this study: 26 adult males, 11 adult females, and 9 female chicks (3–6 days post-hatching) collected from six nests in Novosibirsk during June–July 2016–2021 (54.50° N 83.050° E and 55.090° N 82.950° E). The exact number of individual birds allocated to each experimental group is detailed in Table 1. Birds of two genotypes were analyzed: homozygotes for the standard submetacentric chromosome 1A (PMA1A^S/S^) and heterozygotes carrying the rearranged metacentric variant (PMA1A^S/M^). Homozygotes for the rearranged chromosome (PMA1A^M/M^) were not detected among the sampled birds. No a priori sample size calculation was performed. The sample size was determined by the number of birds that could be captured and sampled from the wild population during the study period to provide a preliminary estimate of the rearrangement frequency. To prevent pseudoreplication in population frequency estimates, only one hatchling per nest was included.

All captured adult birds and hatchlings from the sampled nests were included in the initial population karyotyping. No animals or data points were excluded from the initial karyotyping analysis. For downstream experiments (meiotic analysis, sequencing), birds were selected based on their confirmed PMA1A genotype (heterozygous or homozygous for the wild-type) to enable comparative analysis. All data from these selected birds were included.

The handling and euthanasia of the birds were conducted in compliance with approved national guidelines for laboratory animal care and use. Euthanasia was performed by administering an isoflurane overdose. The study adheres to the ARRIVE guidelines (https://arriveguidelines.org, accessed on 30 May 2025) and was approved by the Animal Care and Use Committee of the Institute of Cytology and Genetics SB RAS (protocol #45/2 of 10 January 2019 and #85 of 15 June 2021).

### 2.2. Fibroblast Cell Cultures and Mitotic Metaphase Chromosomes

To obtain high-quality mitotic chromosomes for karyotyping and FISH we established fibroblast cell culture following the protocol described by Pristyazhnyuk et al. [41]. Mitotic metaphase chromosome spreads were prepared both from fibroblast cell cultures at the second to third passage and from short-term bone marrow cell cultures. The cells were incubated in Dulbecco’s Modified Eagle’s medium with 10 µg/mL colchicine for 2 h at 37 °C. Then the cells were treated with hypotonic solution (0.56% KCl) for 15 min at 37 °C, fixed in methanol-acetic acid (3:1), and spread by air-drying on a microscope slide.

### 2.3. C-Banding, Synaptonemal Complex (SC) Spreading, and Immunostaining

C-banding of mitotic metaphase chromosomes from fibroblast cell cultures was performed using the protocol described by Fernandez et al. [42]. Chromosomes were stained with DAPI.

To visualize synapsis and recombination patterns in meiotic chromosomes, we prepared meiotic chromosome spreads from testes and ovaries by the drying down method [43]. Immunostaining was performed on meiotic spreads using antibodies to SYCP3 to label the SC lateral elements, MLH1 to identify recombination foci, and centromere proteins to mark centromeric regions according to the protocol described by Anderson et al. [44]. We used rabbit polyclonal anti-SYCP3 (1:500; Abcam, Cambridge, UK), mouse monoclonal anti-MLH1 (1:50; Abcam), and human anticentromere (ACA) (1:100; Antibodies Inc., Davis, CA, USA) primary antibodies. The secondary antibodies used were Cy3-conjugated goat anti-rabbit (1:500; Jackson ImmunoResearch, West Grove, PA, USA), FITC-conjugated goat anti-mouse (1:50; Jackson ImmunoResearch), and AMCA-conjugated donkey anti-human (1:100; Jackson ImmunoResearch). Antibodies were diluted in PBT (3% bovine serum albumin and 0.05% Tween 20 in phosphate-buffered saline). A solution of 10% PBT was used for blocking. Primary antibody incubations were performed overnight in a humid chamber at 37 °C; and secondary antibody incubations, for 1 h at 37 °C. Slides were mounted in Vectashield antifade mounting medium (Vector Laboratories, Burlingame, CA, USA) to reduce fluorescence fading.

### 2.4. Chromosome Measurements and Recombination Maps

To compare recombination landscapes between standard and rearranged chromosomes we built the recombination maps. Centromeres were identified by ACA foci. MLH1 signals were only scored if they were localized on SCs. The length of the SC was measured in micrometers and the positions of MLH1 foci in relation to the centromere were recorded using MicroMeasure 3.3 [45]. To generate recombination maps, we divided the length of the SC into equal intervals approximately equal to 1 µm and plotted the proportion of MLH1 foci located in each interval. All results were expressed as mean ± SD; *p* < 0.05 (Mann–Whitney test) was considered statistically significant.

### 2.5. Hybridization Probes and FISH

To identify the chromosome carrying the rearrangement, we used the BAC-clone CH261-36B5 from the CHORI-261 Chicken BAC library [46], kindly provided by D. Griffin (University of Kent), as a probe for FISH on metaphase chromosome spreads. DNA from the BAC clone was extracted with the Plasmid DNA Isolation Kit (BioSilica, Novosibirsk, Russia), amplified using the GenomePlex Whole Genome Amplification Kit (Sigma-Aldrich, St. Louis, MO, USA), and labeled with the GenomePlex WGA Reamplification Kit (Sigma-Aldrich) by incorporating digoxigenin-11-dUTP (Sigma-Aldrich). To reduce nonspecific hybridization, we used the C_0_t-10 DNA from chicken.

To obtain the DNA probe specific to the PMA1A^M^ (further called the PMA1A^M^-specific DNA probe), we microdissected the rearranged chromosome from mitotic metaphase spreads. The spreads were stained by 0.1% Giemsa solution (Sigma-Aldrich) for 5 min at room temperature. The rearranged chromosome was identified as the second-largest metacentric macrochromosome in the karyotype, which has no homologous counterpart. The probe was generated from 15 collected chromosome copies. The amplification of the DNA isolated from the microdissected chromosomal material was carried out with the Whole Genome Amplification 4 (WGA4) kit (Sigma-Aldrich) and 0.1% non-ionic detergent Triton X-100 (VWR Life Science AMRESCO, Solon, OH, USA).

The obtained PCR product was labeled in 20 additional PCR cycles in the presence of fluorochrome-conjugated nucleotide TAMRA-5-dUTP (Biosan, Novosibirsk, Russia). Labeling was carried out using GenomePlex Whole Genome Amplification Reamplification kit (WGA3) (Sigma-Aldrich).

To generate the hybridization DNA probe for CNV8, genomic DNA was extracted from the liver of a female great tit heterozygous for the rearrangement using ExtractDNA Blood&Cells (Evrogen, Moscow, Russia) kit and amplified with primers designed in Primer-BLAST [47] (Supplementary Table S1). The resulting PCR products were labeled in 20 additional PCR cycles in the presence of fluorochrome-conjugated nucleotide Tamra-5-dUTP (Biosan).

FISH on metaphase chromosomes was performed according to a standard protocol [48]. Chromosomes were counterstained with DAPI dissolved in Vectashield antifade solution (Vector Laboratories).

### 2.6. Microscopic Analysis

Images of DAPI-stained metaphase chromosomes and SC spreads after immunostaining and FISH were captured using a CCD-camera installed on an Axioplan 2 compound microscope (ZEISS, Jena, Germany) equipped with CHROMA filter sets (ZEISS) using ISIS4 (METASystems GmbH, Altlußheim, Germany). All microscopy studies were carried out at the Center for Microscopic Analysis of Biological Objects of SD RAS (Novosibirsk, Russia). Images were processed using Corel PaintShop Pro X6 (Alludo, Ottawa, ON, Canada).

### 2.7. Library Preparation and Sequencing

Hi-C libraries were prepared from fibroblast cell cultures derived from one homozygous (PMA1A^S/S^) and one heterozygous (PMA1A^S/M^) female great tits using DpnII for DNA fragmentation and the KAPA HyperPlus kit (Basel, Switzerland) according to manufacturer’s protocol as previously described [49]. The Hi-C libraries were sequenced by BGI on the DNBSEQ platform with a paired-end 150 bp layout.

For whole-genome sequencing (WGS), DNA was extracted from the livers of two male great tits heterozygous for the rearrangement in PMA1A and two female great tits homozygous for the wild-type chromosome using the standard phenol-chloroform method. The WGS libraries were prepared and sequenced by BGI on the DNBSEQ platform with a paired-end 150 bp layout, generating approximately 30× coverage per sample.

For Oxford Nanopore sequencing, DNA was isolated from the fibroblast cell culture derived from female great tits heterozygous for the rearrangement in PMA1A using the standard phenol-chloroform method. The library was prepared using the recommended protocol for LSK114 kit from the manufacturer (Oxford Nanopore, Oxford, UK). Sequencing was performed on a MinION device with the FLO-MIN114 Flow Cell (Oxford Nanopore), yielding an approximately 10× genome-wide coverage.

### 2.8. Hi-C Contact Map Construction

Hi-C data analysis was performed using a custom version of Juicer tools v.1.22.01 [50] with a statistics module modified as described in Ma et al. [51]. Hi-C reads were aligned to the NCBI reference assembly Parus_major1.1 (GCF_001522545.3) [35]. Manual assembly curation for chromosome 1A was performed using Juicer assembly tools v.1.22.01 [50]. The final curated version of the assembly was designated Parus_major1.1_p2. The correspondence of coordinates between the Parus_major1.1_p2 and Parus_major1.1 assemblies is provided in Supplementary File S1.

### 2.9. CNV Analysis

WGS data were used to identify CNVs and assess their amplification based on read depth analysis. Reads were aligned to the great tit reference genome assembly Parus_major1.1 GCF_001522545.3 (Supplementary Table S2) and to its updated version Parus_major1.1_p2 (Supplementary Table S3) using BWA-MEM v.0.7.19-r1273 with default parameters [52]. Coverage was assessed using samtools depth [53]. CNV regions were identified as genomic intervals showing consistent differences in read depth between heterozygotes and homozygotes. Their abundance was then estimated relative to baseline coverage. To establish a baseline for normal diploid coverage, the average sequencing depth was calculated across large genomic regions of PMA1A outside the CNVs. For each CNV interval, the mean read depth was then measured and compared with this baseline to estimate the total copy number in each sample. The amount of additional DNA introduced by each CNV was calculated as the product of its length and the number of copies exceeding the normal diploid state. The cumulative size of CNVs in each sample was calculated as the sum of additional sequence lengths across all CNVs.

To detect CNVs related to rearranged PMA1A, which are not represented in the reference assembly, we analyzed Hi-C data from homozygous and heterozygous birds using a combination of exploratory analyses via genomic data visualization tools and custom scripts. Read pairs in which one mate was mapped to the PMA1A while the other mate remained unmapped were extracted and processed with Jellyfish v.2.3.1 [54] to estimate k-mer abundance. We visualized k-mer frequency scatterplots for k in range 80…149 (a representative example is shown in Supplementary Figure S1) and selected k-mers significantly enriched in the heterozygote for subsequent analyses. Visual inspection indicated that most of the selected k-mers were redundant, representing shifted subsequences of the same sequence. To assemble the final sequence, we built the consensus using Unipro UGENE v40.1 [55] with MUSCLE alignment backend.

To analyze co-localization patterns of CNVs, we aligned CNV sequences to the Nanopore reads using blastn v2.11.0+ [56] with parameters: -word_size 7 and -dust no. Short (<200 bp) and low identity (<80%) fragments were filtered out. To eliminate redundant/nested HSPs, for each read, hits were sorted by alignment length and accepted iteratively. A new hit was discarded if ≥80% of its length overlapped an already accepted hit on the read. Adjacent or gap-separated hits were considered distinct.

### 2.10. Reconstruction and Analysis of the FAM118A-Homologous Locus

To test for the presence of *FAM118A*-homologous sequences in the great tit, Oxford Nanopore reads were mapped to the predicted *FAM118A* coding sequence of the blue tit (*Cyanistes caeruleus*) (NCBI RefSeq accession XP_023774836.1) using minimap2 v.2.29-r1283 [57]. Mapped reads were extracted for downstream analyses. To estimate the number and organization of CNV8 copies within *FAM118A*-homologous sequences, the CNV8 consensus was aligned to Nanopore reads using minimap2 v.2.29-r1283 [57].

To reconstruct the structure of the *FAM118A*-homologous locus, we selected Nanopore reads containing both flanking regions and a single CNV8 unit. These reads were aligned using MAFFT v7.525 [58] and a consensus sequence was generated using AliView 1.30 [59]. The predicted FAM118A protein sequence of the blue tit (XP_023774836.1; 357 aa, corresponding to the canonical human isoform) was aligned to the great tit consensus using miniprot v0.13-r248 [60].

Read-depth profiles across the *FAM118A*-homologous region and adjacent CNVs were calculated based on short-read alignments using BWA as described above. Single-nucleotide variants introducing premature stop codons within exon 2 were identified by aligning both long and short reads to the consensus sequence and inspecting the alignments in IGV to verify the affected codons.

### 2.11. Population Frequency Estimates and Statistical Analyses

Rearranged chromosome PMA1A frequencies were estimated separately for males and females to account for possible sex-specific differences. The sampling variance of the frequency estimate *p* was calculated as *p*(1 − *p*)/N, where N equals the number of individuals for genotype frequencies and the number of PMA1A chromosomes for chromosome frequencies. Differences between sexes, populations and deviations from Hardy–Weinberg equilibrium (HWE) (which assumes random mating and no evolutionary forces) were tested using chi-square (χ^2^) statistics. All statistical analyses were performed using the Statistica 6.0 software package (StatSoft Inc., Tulsa, OK, USA).

## 3. Results

### 3.1. Mitotic Chromosome Analysis Reveals PMA1A Heteromorphism in Natural Populations of the Great Tit

**Figure 1:**
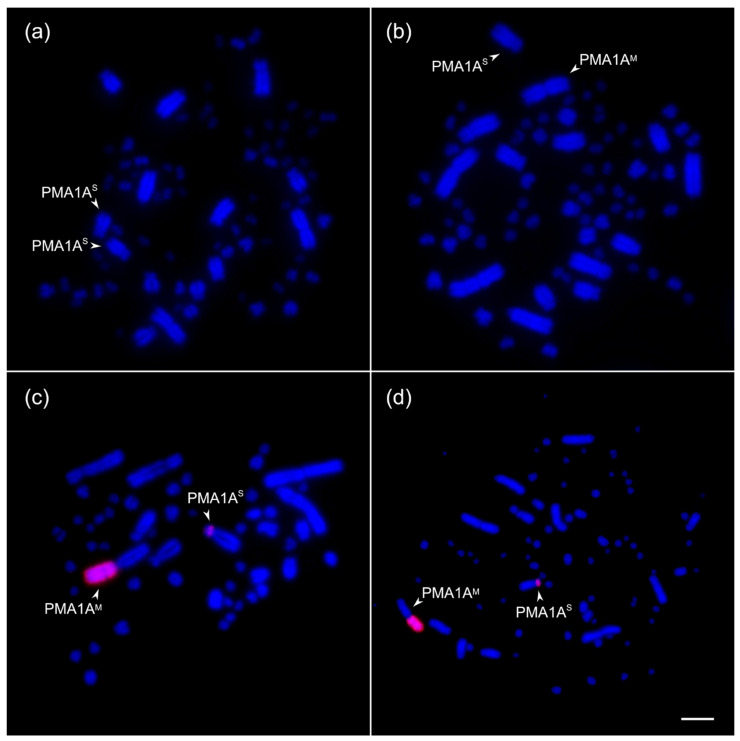
Metaphase chromosomes from fibroblast cultures of a homozygous (PMA1A^S/S^) male (**a**) and a heterozygous (PMA1A^S/M^) female great tit (**b**–**d**) after staining with DAPI; FISH with the PMA1A^M^-specific DNA probe (red) (**c**), and FISH with the CNV8-specific hybridization probe (red) (**d**). Scale bar—5 µm.

During routine karyotyping of great tits collected in Novosibirsk (Western Siberia), we detected several birds carrying a heteromorphic pair of chromosomes. One homolog was a large submetacentric chromosome, which corresponded to the standard chromosome 1A in length (2.6 ± 0.3 μm, 4th largest) and centromeric index (0.27 ± 0.07), whereas the other homolog was a metacentric chromosome with significantly greater length (4.0 ± 0.5 μm) and centromeric index (0.49 ± 0.03) (Mann–Whitney test, *p* < 0.05) (Figure 1a,b). One arm of this homolog was C-positive, indicating chromatin condensation typical of heterochromatic regions (Supplementary Figure S2).

Fluorescence in situ hybridization (FISH) with a universal avian BAC clone for chromosome 1A (CH261-36B5) from the CHORI-261 Chicken BAC library [46] produced a strong signal on the short arm of the standard submetacentric chromosome and on one of the arms of the metacentric chromosome (Supplementary Figure S2). We designated these variants as PMA1A^S^ and PMA1A^M^ variants, respectively.

To determine the origin of PMA1A^M^, we microdissected it, prepared the whole-chromosome paint probe, and hybridized it to mitotic metaphase chromosomes of PMA1A^S/M^ birds (Figure 1c). We detected a strong hybridization signal completely covering one arm of PMA1A^M^. In PMA1A^S^, the probe marked the short arm. This pattern indicates the presence of multiple repetitive sequences in the short arm of PMA1A^S^. Amplification of these repeats apparently caused elongation of the short arm of PMA1A^M^. Hereafter, we refer to this arm of the rearranged metacentric chromosome as the short arm. No signals of the microdissected probe were detected on the long arms of either PMA1A^S^ or PMA1A^M^, most likely due to an overrepresentation of short-arm repeats in the hybridization probe.

### 3.2. The Rearranged Chromosome Is Present in a Low Frequency in the Siberian Population

Using live traps, we captured 11 female and 26 male adults from the urban population of Novosibirsk. Additionally, we collected nine female hatchlings from six nests, containing 2, 3, 1, 1, 1 and 1 hatchlings, respectively. All specimens were karyotyped by SC or metaphase chromosome analysis. Karyotyping revealed that both hatchlings from the first nest were standard homozygotes PMA1A^S/S^, while all three hatchlings from the second nest were heterozygotes PMA1A^S/M^. To prevent pseudoreplication, we included only one hatchling per nest when estimating the population frequency of the PMA1A^M^ chromosome.

The frequency of the heterozygotes for the rearranged chromosome was 0.24 ± 0.11 in females and 0.15 ± 0.07 in males (Table 2). This sex difference was not statistically significant (χ^2^ = 0.8, *p* = 0.4), so we report a sex-averaged frequency of 0.19 ± 0.06. Cytotype frequencies did not deviate significantly from HWE expectations (χ^2^ = 0.5, *p* = 0.5).

### 3.3. Chromosome Synapsis and Recombination Patterns Indicate Inversion and Additional Genetic Material in PMA1A^M^

**Figure 2:**
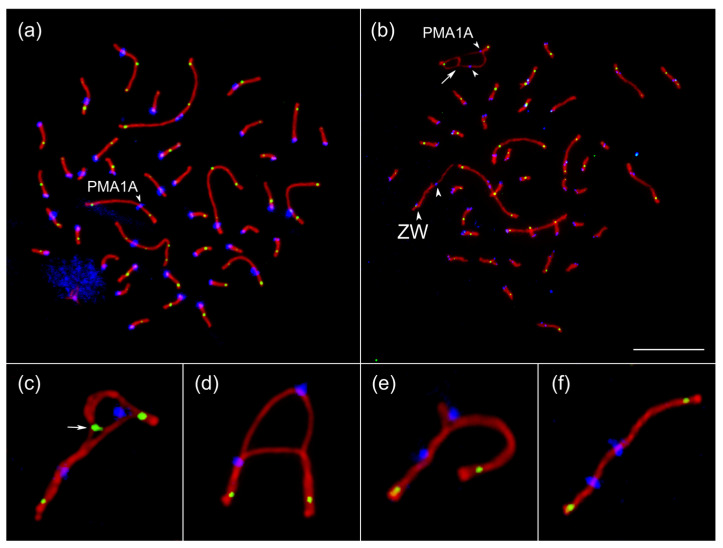
Synaptonemal complex spreads of the great tit. Pachytene spermatocyte of the PMA1A^S/S^ homozygote (**a**) and oocyte of the PMA1A^S/M^ heterozygote (**b**) after immunolocalization of SYCP3 (red), MLH1 (green), and centromeric proteins (blue). Arrowheads point to the centromeres of the PMA1A bivalents and the ZW sex bivalent (identified by the heteromorphic SC and misaligned centromeres). The arrow points to the interstitial homologously paired region. (**c**–**f**) Synaptic configurations of the PMA1A^S/M^ bivalent: inversion loop (**c**), D-loop (**d**), hairpin (**e**), and linear bivalent (**f**). The arrow points to the MLH1 focus at the base of the inversion loop (**c**). Scale bar—10 µm.

To detect chromosome rearrangements underlying PMA1A heteromorphism, we analyzed patterns of chromosome synapsis and recombination during male and female meiosis in PMA1A^S/M^ heterozygotes compared with PMA1A^S/S^ homozygotes (Figure 2). We performed immunolocalization of SYCP3 to visualize the lateral elements of the SC, MLH1 to mark recombination nodules, which can occur only in the regions of homologous pairing, and centromere proteins to visualize the centromeres. This approach allowed us to reveal heteromorphic synaptic configurations and to distinguish regions involved in homologous synapsis from those paired non-homologously through synaptic adjustment, based on the presence or absence of MLH1 foci.

In all pachytene spermatocytes and oocytes of heterozygous birds, we observed a large heteromorphic bivalent with two distinct, non-aligned centromeric signals (Figure 2b–f). We identified four types of synaptic configurations of the PMA1A^S/M^ bivalent: an inversion loop (Figure 2c); a D-loop (Figure 2d) or hairpin (Figure 2e) formed by one of the lateral elements of the SC; and linear bivalents (Figure 2f).

Inversion loops unequivocally indicate heterozygosity for an inversion. The configurations of the inversion loops (specifically, the involvement of the long arms in non-collinear homologous synapsis) and the position and extent of homologous contacts in the partially synapsed, unadjusted configurations (Figure 2b) demonstrate that the inversion is located in the long arm of PMA1A and spans almost its entire length. The relative positions of the centromere signals on the lateral elements of the PMA1A^S^ and PMA1A^M^ SCs indicate that the inversion does not include the centromere and is therefore paracentric. If the centromere were included within the inversion, its signal on the rearranged chromosome would be displaced toward the proximal end of the bivalent.

The presence of D-loops and hairpins indicates that the PMA1A^M^ homolog contains additional genetic material constituting at least 30% of the PMA1A^S^ length. Linear bivalents with two non-aligned centromeric signals apparently result from equalization of the SC lateral elements, through stretching of the PMA1A^S^ element and compaction of the PMA1A^M^ element. We suggest that bivalents with D-loops, hairpins, and linear configurations represent successive stages of equalization, resulting in non-homologous synapsis typical for the synaptic adjustment of heteromorphic autosomes [61,62,63] or sex chromosomes [64,65,66].

SC analysis confirms that complex rearrangement in PMA1A involves an inversion in the long arm and an amplification of the genetic material in the short arm. To roughly assess the borders of this rearrangement in heterozygotes, we analyzed the distribution of MLH1 foci, which can only occur in the homologously paired regions.

**Figure 3:**
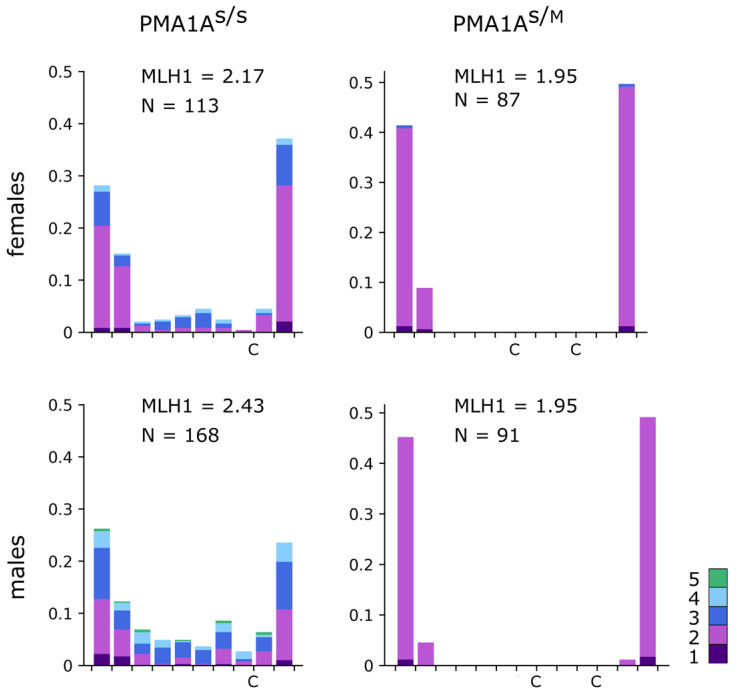
Distribution of MLH1 foci, marking recombination nodules, along the PMA1A^S/S^ and PMA1A^S/M^ bivalents in oocytes and spermatocytes. The *x*-axis shows the relative position of MLH1 foci with respect to the centromere (C), with bins of 1 µm. The *y*-axis indicates the frequency of MLH1 foci in each bin. Colors indicate bivalents with 1–5 MLH1 foci per bivalent.

In PMA1A^S/S^ homozygote, MLH1 foci distribution was typical for submetacentric chromosomes of comparable length in birds and other vertebrates (Figure 3) [67,68]. Prominent peaks were observed near both telomeres, with a smaller secondary peak in the middle of the long arm, and a relatively even distribution across the remaining regions.

In heterozygotes, we analyzed the MLH1 distribution in the linear bivalents, which resulted from synaptic adjustment and length equalization between homologs (Figure 2f). In both males and females, the distribution was bimodal, with most foci located close to the telomeres, indicating that homologous recombination was blocked along most of the PMA1A^S/M^ bivalent. The recombination pattern thus supports a large inversion covering most of the long arm. However, given the uneven involvement of chromatin in the synaptonemal complex [69], especially in the presence of synaptic adjustment, quantitative estimates of the inversion boundaries cannot be inferred from this analysis.

### 3.4. Hi-C Analysis Defines the Boundaries of the Inversion in PMA1A^M^

To delineate the boundaries of the inversion and investigate the nature of the amplification, we performed Hi-C sequencing of one homozygous (PMA1A^S/S^) and one heterozygous (PMA1A^S/M^) female great tit and generated Hi-C contact maps by aligning the reads to the reference genome Parus_major1.1 (GCF_001522545.3).

The Hi-C map of a PMA1A^S/S^ homozygote revealed contact patterns indicative of multiple chromosomal rearrangements or potential misassemblies in the reference genome (Supplementary Figure S3). Several regions lacked interactions with adjacent loci but showed strong contacts with distant regions. The presence of misassemblies in the great tit reference genome seems plausible, as it is based on short-read Illumina sequencing scaffolded with a genetic linkage map [35]. We manually curated the Hi-C data using the Juicebox tool [70], resulting in a refined version of the PMA1A assembly (Parus_major1.1_p1).

**Figure 4:**
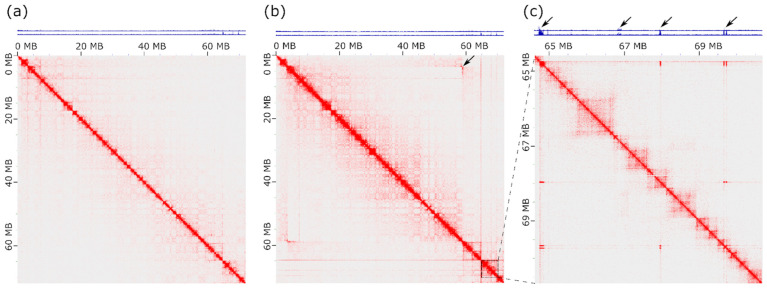
Hi-C contact maps of PMA1A in the PMA1A^S/S^ homozygote (**a**) and the PMA1A^S/M^ heterozygote (**b**), with a zoom-in of the distal region containing CNVs (**c**). Maps were generated using the updated PMA1A assembly Parus_major1.1_p1. Hi-C reads coverage tracks are shown in blue above each map. Data presented at 100 kb resolution. The arrow in (**b**) marks the butterfly-like contact pattern characteristic of a chromosomal inversion. Arrows in (**c**) highlight contact-dense segments with multiple interactions to distant genomic regions and elevated coverage.

Using the refined assembly, we reconstructed Hi-C contact maps of PMA1A for homozygotes and heterozygotes (Figure 4a,b). In heterozygotes, we observed a butterfly-like contact pattern characteristic of a large inversion (Figure 4b) [71]. This pattern persisted after flipping the inverted region, confirming the heterozygous state of the inversion.

From the Hi-C data, we determined the inversion breakpoints at approximately 3503 ± 5 kb and 56,873 ± 5 kb in Parus_major1.1 assembly (corresponding to 3412 ± 5 kb and 56,942 ± 5 kb in Parus_major1.1_p1), with an estimated inversion size of 55.1 Mb. The size and position of the inversion are consistent with cytogenetic evidence indicating that the inversion involves only the long arm of PMA1A, which constitutes ~75% of its length.

### 3.5. CNV Analysis Reveals Extensive Amplification of a Short Repetitive Sequence

In addition to the inversion pattern, the Hi-C contact map of the heterozygous bird displayed patterns characteristic of CNVs, which were not observed in the wild-type homozygote. These regions appeared as contact-enriched segments that interacted not only with neighboring loci but also with multiple distant genomic regions (Figure 4b,c). The coverage plots confirm their high copy number.

To validate the presence of CNVs and estimate their abundance, we conducted short-read whole-genome sequencing of two homozygous females and two heterozygous males. Read depth analysis of short-read alignments to the reference PMA1A assembly identified seven CNV regions located within 63–68 Mb (Supplementary Table S2). The lengths of these regions ranged from 3 to 97 kb. Both homozygous females carried two copies of CNV1–6, consistent with the diploid state, and approximately 40 copies of CNV7 (about 20 copies per homolog). Heterozygous males carried about 20 copies of CNV1–3, CNV5, and CNV6 and up to 400 copies of CNV4 and CNV7.

Based on the lengths of CNV regions and their approximate copy numbers, we estimated that the rearranged chromosome exceeds the reference PMA1A by approximately 9.5 Mb, which is about 13% of its length. However, our cytological estimates indicate a larger increase in length, ranging from 30% based on SC analysis to 50% based on metaphase chromosome measurements. This discrepancy may be due to unique or repetitive sequences present in PMA1A^M^ that are missing from the reference genome assembly.

To find such repetitive sequences, we developed a computational pipeline and applied it to Hi-C sequencing data from homozygous and heterozygous birds. In the first step, we extracted read pairs in which one mate aligned to the reference PMA1A while the other remained unmapped, thereby enriching for sequences adjacent to assembled PMA1A regions. In the second step, we compared k-mer abundances between the homozygous and heterozygous Hi-C datasets and selected those significantly enriched in the heterozygous case. A subset of overlapping k-mers (Supplementary Figure S1) was then manually assembled into a 630 bp contig.

BLAST analysis confirmed that this sequence is absent from the Parus_major1.1 reference genome assembly. We designated this contig CNV8 and incorporated it into the refined PMA1A assembly. Read-depth analysis of WGS alignments to the refined PMA1A demonstrated that CNV8 is massively amplified, with copy numbers of 7000–9000 in homozygous and ~50,000 in heterozygous birds.

To analyze the distribution of CNV8, we generated a FISH probe and hybridized it to mitotic metaphase chromosomes of a heterozygous bird. The probe produced strong signals on the short arms of PMA1A^S^ and PMA1A^M^ (Figure 1d). The distinct signal on the short arm of PMA1A^S^ confirms the high copy number of this repeat in wild type PMA1A. In PMA1A^M^, the probe labeled the entire short arm, consistent with extensive amplification and potential tandem organization of this repeat.

To explore the genomic arrangements of CNVs, we performed low-coverage Nanopore sequencing and aligned the CNVs to individual reads. We found that CNV1–3 consistently co-localized within the same reads and likely formed a contiguous block. However, they were never observed together with other CNVs. The CNV1–3 block showed heterogeneous and structurally complex organization, characterized by fragmented alignments, variable percent identity, and segments occurring in opposite orientations (Supplementary Table S4). CNV4 and CNV7 often appeared together within the same reads, suggesting they constitute a separate cluster. CNV8 displayed a distinct organization, predominantly forming long head-to-tail tandem arrays of 630 bp units, which extended up to ~80 kb (the maximum span detectable within single Nanopore reads) and showing high coverage and sequence identity (Supplementary Table S4).

### 3.6. Long-Read Analysis Identifies Large-Scale Amplification of the FAM118A Locus

To identify the origin of the massively amplified CNV8 repeat, we queried the 630 bp contig against the NCBI nucleotide collection (core_nt) using BLASTn. The search returned significant alignments (>600 bp, 87–88% identity) to chromosome assemblies of several passerine species and to predicted *FAM118A* mRNAs. A 221–224 bp segment of CNV8 aligned with the complete third (or fourth, depending on the isoform) exon of these predicted mRNAs with 93–98% identity. No match was found in the Parus_major1.1 genome assembly, indicating that CNV8 corresponds to a *FAM118A*-derived fragment absent from the current great tit reference.

*FAM118A* is a conserved protein-coding gene of unknown function, located within a syntenic block corresponding to human chromosome 22q13.31 and conserved across birds. In several passerine genomes, this locus is annotated as predicted *FAM118A*, underscoring its conservation; its apparent absence from the great tit reference genome likely reflects incomplete assembly rather than actual gene loss. To test whether *FAM118A* is present in the great tit, we mapped ONT long reads to the predicted *FAM118A* sequence of the blue tit (*Cyanistes caeruleus*). The alignment produced almost 100-fold higher coverage than the genome-wide average, confirming the presence of multiple copies of *FAM118A*-homologous sequence in the heterozygous individual. Further analysis revealed that these *FAM118A* copies contain varying numbers of tandemly repeated CNV8 copies.

We generated a consensus sequence from ONT reads containing a single CNV8 unit within the *FAM118A*-homologous region (Supplementary File S2). Alignment of this consensus and CNV1-7 back to ONT reads indicated that its terminal portion overlaps CNV7, while the proximal end neighbors CNV4. We extended the consensus to fully include CNV7 and incorporated it into the refined assembly Parus_major1.1_p1, generating the final version Parus_major1.1_p2. Notably, this placement reflects the predominant configuration observed in ONT reads from heterozygotes, while the position and organization of the *FAM118A*-homologous locus may differ in the wild-type PMA1A^S^.

**Figure 5:**
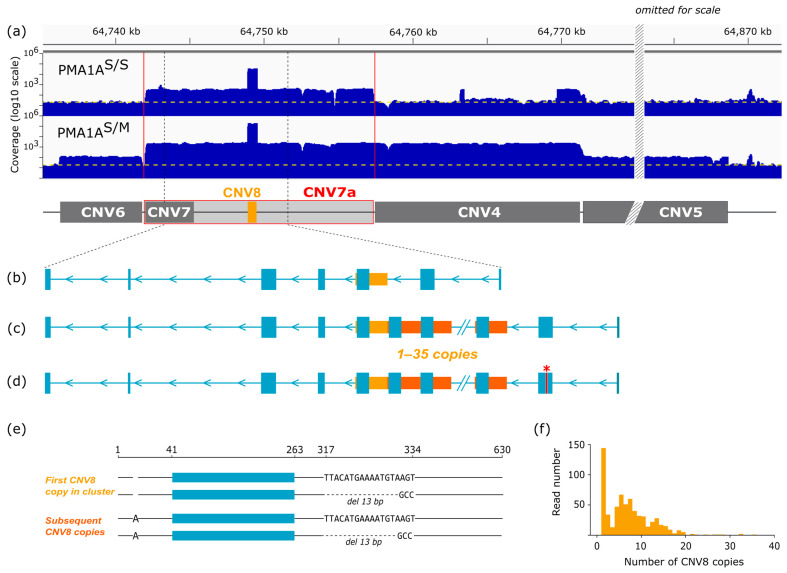
Amplification and structural organization of the *FAM118A*-homologous sequence in the great tit. (**a**) Read-depth profiles across the genomic region containing the *FAM118A*-homologous sequence and adjacent CNVs in homozygotes (PMA1A^S/S^) and heterozygotes (PMA1A^S/M^). Coverage is plotted on a log_10_ scale. The yellow dashed lines mark the genome-average coverage baseline (~30×). The vertical red lines mark the edges of the inserted consensus sequence CNV7a. (**b**–**d**) Structural organization of the *FAM118A*-homologous sequence. Blue rectangles represent *FAM118A* exons; yellow and orange boxes denote CNV8 copies; arrows indicate repeat orientation. (**b**) Structure of the consensus great tit *FAM118A*-homologous sequence derived from the miniprot alignment of the 357-aa predicted blue tit FAM118A protein (corresponding to the canonical human isoform). (**c**) Schematic organization of the CNV8 tandem array within the *FAM118A*-homologous sequence. (**d**) Variant configuration showing a copy containing a premature stop codon (red asterisk) within exon 2. (**e**) CNV8 sequence variants within the *FAM118A*-homologous sequence. Blue rectangles represent *FAM118A* exons. (**f**) Distribution of ONT reads containing different numbers of CNV8 copies within the *FAM118A*-homologous sequence.

We designated the extended consensus sequence including CNV7 as CNV7a and re-mapped short reads from heterozygotes and normal homozygotes to the final assembly. We found that the *FAM118A*-homologous locus is present in approximately 40 copies in homozygotes (PMA1A^S/S^) and in about 400 copies in the heterozygotes (PMA1A^S/M^) (Supplementary Table S3; Figure 5a). Considering other CNVs, including the massively amplified CNV8, we estimated that PMA1A^S^ is about 2.5 Mb larger than the reference PMA1A assembly, while PMA1A^M^ exceeds it by approximately 40 Mb, which explains the cytologically observed increase in the length of the rearranged chromosome (Supplementary Table S3).

To evaluate whether the consensus *FAM118A* sequence of great tit represents an intact gene, we aligned the predicted blue tit FAM118A protein (357 aa, corresponding to the canonical human isoform) to the consensus. The alignment revealed a full coding sequence, with all exons in the expected order and reading frame (Figure 5b–d) (Supplementary File S3). Analysis of long-read alignments showed that approximately 40% of *FAM118A*-homologous reads contained a premature stop codon within the second exon. Short-read data confirmed this result, revealing that about 60% of reads from heterozygotes carried the same mutation, whereas no stop codon was detected in reads from normal homozygotes.

The third exon of *FAM118A* is entirely embedded within CNV8. Analysis of ONT reads spanning both flanking regions of the CNV8 cluster showed that the number of CNV8 copies within the *FAM118A* locus in heterozygotes reaches at least 35 (Figure 5c,d,f). In most cases, the first CNV8 copy differed from the subsequent ones, although these differences were located outside the exon sequence (Figure 5e). Among reads containing a single CNV8 unit and both flanking regions including exon 2, 16% did not contain the premature stop codon.

We could not directly confirm the amplification of CNV8 within the *FAM118A* locus in normal homozygotes due to the lack of ONT reads. However, the higher coverage of CNV8 compared to the rest of the locus (Figure 5a) and the presence of multiple short reads spanning junctions between adjacent CNV8 copies suggest that this amplification also occurs within *FAM118A* copies in normal homozygotes.

### 3.7. Siberian and European Great Tits Share a Complex Rearrangement in PMA1A

A similar complex rearrangement on PMA1A was identified in European populations of the great tit [40]. Based on PCA, SNP-wise F_ST_ and heterozygosity patterns of SNP-typed birds, da Silva et al. [40] inferred that ~5% of the Netherlands population were heterozygous for a large inversion on PMA1A (~64 Mb; >90% of the chromosome length). Heterozygotes for this inversion were also found among resequenced individuals from France and Belgium. Using read-depth analysis of these birds, da Silva et al. [40] detected a previously described CNV complex (CNVR “2802”; ~64.83–67.67 Mb; [72]) at the downstream inversion breakpoint and additional upstream CNVs (Table 3). They estimated that together these CNVs may account for up to 3.5 Mb of increase in the rearranged PMA1A compared to the reference chromosome.

We infer that these Siberian and European variants of PMA1A represent the same complex rearrangement. This conclusion is supported by the coincidence of the proximal inversion breakpoint and by the detection of the same CNV sequences at comparable levels of amplification, including CNV1–3 comprising the CNVR “2802” complex and the upstream copy number gains designated CNV4 and CNV5 in our study.

## 4. Discussion

We discovered a complex rearrangement of the chromosome 1A in the Siberian population of the great tit that combines a large paracentric inversion (~55 Mb) on the long arm with a dramatic gain of sequence (>30 Mb) on the short arm. Hi-C and cytogenetic data delineate the inversion boundaries and show that recombination is suppressed along almost the entire chromosome. The expansion of the short arm resulted from large copy-number gains of several genomic regions, including amplification of a segment harboring a putative *FAM118A* gene that carries an internal short tandem repeat array.

Presumably the same complex structural variant of chromosome 1A with a shared inversion and CNV characteristics (Table 3) has been previously described in European populations of the great tit [40]. Discrepancies in the reported inversion size (~65 Mb vs. ~55 Mb in our study; Table 3) and estimated chromosome length gain (~3.5 Mb vs. ~30 Mb in our study) apparently reflect methodological differences and incompleteness of the reference assembly, although the difference in copy-number variation between the two geographically distant populations cannot be excluded.

The inversion size in the European dataset is likely overestimated because suppressed recombination in the distal part of PMA1A (Figure 3) yields a heterozygosity pattern mimicking that expected under inversion. Suppression of recombination in this region, which is supported by LD patterns in the European population [40] and cytological data from Siberian great tits (Figure 3), may be explained by local heterochromatinization, spatial pairing constraints imposed by the inversion in the long arm, and/or additional small inversions that further hinder homology search [40]. Most of the short-arm increase was apparently missed by read-depth analysis of the European heterozygotes, since the reference assembly lacks most of the *FAM118A* locus harboring CNV8, the major contributor to the PMA1A size burst.

In the Siberian population, we detected a moderate amplification of *FAM118A* (and CNV8) in homozygotes for the normal PMA1A variant (Figure 5a). Whether a similar amplification is present in wild-type European great tits remains to be determined. However, da Silva et al. [40] reported that 11.3% of 1921 normal homozygotes carried CNVs (“2802”).

Based on the available data, it is not possible to determine the cause and mechanism of the inversion formation. However, an amplification of the *FAM118A* locus (Siberian population) and CNV complex “2802” (European population) in wild-type homozygotes suggests that the structural changes in the short arm preceded the origin of inversion in the long arm and may have facilitated its formation. The inversion itself, with its distal breakpoint located apparently near the centromere, may suppress recombination along the most part of the short arm due to spatial constraints leading to the further accumulation of repetitive sequences.

Finding the same complex rearrangement in Siberian and European populations separated by more than 5500 km implies a shared ancient origin. Da Silva et al. [40] speculated that the European inversion might have arisen more than 10^5^ generations ago. If this estimate is correct, the polymorphism must have originated before the end of the last glacial period (~12,000 years ago). The great tit as a species shows extremely shallow genetic differentiation, likely resulting from a drastic range contraction and population reduction during the last glacial maximum [73,74], followed by rapid recolonization from a single refugium and demographic expansion. No isolation-by-distance has been detected across Northern Eurasia [75,76,77]. Therefore, the currently available population genetic data cannot tell us where the PMA1A^M^ variant occurred.

The frequency of heterozygotes for the rearrangement is significantly higher in Siberia (0.19 ± 0.06) than in Western Europe (0.05 ± 0.00; χ^2^ = 17.8, *p* < 0.0001), while no homozygotes were observed in either population. Both populations conform to Hardy–Weinberg equilibrium for the PMA1A chromosome variants (χ^2^ = 0.5, *p* = 0.5 for Siberian population and χ^2^ = 1.6, *p* = 0.2 for the European population). Given these frequencies of the heterozygotes and sample sizes, homozygotes for the rearrangement might have been missed in both studies.

However, in follow-up research, da Silva [78] reported recessive lethality of the rearranged PMA1A variant. Crosses between heterozygotes produced no inversion homozygotes and showed 20% lower hatching success compared with crosses between heterozygotes and normal homozygotes and between normal homozygotes themselves. This study also demonstrated preferential transmission of the rearranged variant through males (≈67% instead of 50% expected under Mendelian law).

Taken together, these data suggest that the relatively low frequency of the rearranged PMA1A variant might be maintained by a balance between lethal homozygosity and transmission bias, although the molecular basis of both effects remains unknown.

In our study, we found that the enlarged short arm of the PMA1A^M^ contains a massive amplification of a genomic region encompassing a complete sequence homologous to the *FAM118A* gene. *FAM118A* (family with sequence similarity 118 member A) is a conserved but poorly characterized protein-coding gene [79], which is missing from the current great tit reference genome assembly. Recent study in humans has shown that this protein belongs to a previously uncharacterized SIRim (SIR-related immune) subfamily of immune proteins conserved across diverse eukaryotic lineages, and that its paralog *FAM118B* plays a pivotal role in innate immune activation through the Toll-like receptor (TLR) pathway [80].

In the wild-type PMA1A^S^ chromosome, the *FAM118A*-homologous region occurs in approximately 20 copies, while in the rearranged PMA1A^M^ variant it is further amplified roughly twentyfold. Whether these multiple copies are functional remains unknown. However, alignment of the great tit consensus sequence with predicted FAM118A orthologs from related passerine species suggests that at least a subset of the copies may retain an intact open reading frame and could potentially encode functional proteins.

In contrast, more than half of the *FAM118A* copies in the rearranged chromosome apparently carry a premature stop codon within the second exon, which may result in truncated proteins or nonfunctional transcripts. Further analyses are required to determine whether these defective copies exert any physiological effects or contribute to the recessive lethality associated with the PMA1A^M^ rearrangement.

The functional relevance of the massive amplification of the 630 bp tandem repeat within the *FAM118A* locus, designated CNV8 in our study, also remains unclear. Analysis of long ONT reads from heterozygotes showed that *FAM118A* copies contain up to 35 tandemly arranged CNV8 units, as confirmed by reads spanning both flanking regions. We also identified long reads consisting entirely of CNV8 arrays extending up to ~80 kb. However, it is unclear whether these long arrays are located within the *FAM118A* gene. In total, the rearranged chromosome is estimated to contain approximately 50,000 copies of this repeat, which, together with other less amplified CNVs, explains a 1.5-fold increase in the length of the PMA1A^M^ chromosome.

The CNV8 sequence completely encompasses exon 3 of *FAM118A*. In part of *FAM118A* copies from PMA1A^M^ that carry a premature stop codon in exon 2, the CNV8 array lies downstream of the truncation site and is unlikely to be translated, although it may still affect mRNA processing, transcript stability, or chromatin organization. At the same time, other *FAM118A* copies in the rearranged chromosome, which retain an intact reading frame and contain multiple repeats of exon 3 within the amplified CNV8 region, may still be functional.

Putatively functional *FAM118A* copies in wild-type homozygotes also appear to harbor an amplification of CNV8, as indicated by the markedly higher coverage of this sequence. However, the absence of ONT reads from normal homozygotes did not allow us to directly check this hypothesis. Whether these variants produce full-length transcripts that include the expanded exon 3, or whether the CNV8 arrays have acquired a noncoding regulatory function remains to be tested.

Future studies should also aim to determine whether any adaptive advantage in natural populations contributes to the maintenance and spread of this complex inversion–amplification PMA1A rearrangement in addition to its presumed transmission bias. Given the suppression of recombination across most of the chromosome, it is plausible that the rearrangement acts as a supergene maintaining a co-adapted haplotype block. Such structural variants are known to facilitate local adaptation in many taxa by linking together alleles that function optimally under specific environmental conditions [81,82].

The structure, genetic content and transmission peculiarity of the complex chromosome rearrangement found in European and Siberian populations are somewhat reminiscent of those reported for a complex chromosomal rearrangement on chromosome 1 of the house mouse [83,84,85]. The rearranged chromosome of the house mouse also contains a large inversion and a massive amplification of the gene *SP100* involved in anti-viral response through activation by interferons [86,87,88]. This rearranged chromosome variant shows a strong meiotic drive in females [84,89,90]. It is not beyond reason to draw attention to the possibility of a molecular convergence between these two species.

The relatively high frequency of the PMA1A^M^ variant in the Siberian population raises the possibility that it may be favored under particular ecological conditions, such as colder climate, differences in migratory behavior, or reproductive timing. In accordance with this view, we have an anecdotal observation that, however, should be taken with caution. At the very beginning of the breeding season, we sampled four adult males. Two males with large testes and mature sperm were heterozygotes, while the other two males were homozygous for the standard PMA1A and had very small testes. If this observation is confirmed, it might indicate a selective advantage of the heterozygotes due to the early entering into the breeding season and higher chances to produce two broods within a single breeding season.

Indeed, several genes located within inverted region on PMA1A (*ASB15*, *KCNC2*, *NAV3*, *SLC6A15*, *MGAT4C*) were among the candidate loci identified by Stonehouse et al. [38] as putatively associated with climatic adaptation. In the European population, however, these assumptions were not supported. No association between the presence of the rearrangement and reproductive traits such as laying date, clutch size, or number of fledged chicks was found [78].

Altogether, this complex inversion–amplification rearrangement of PMA1A represents one of the most striking cases of large-scale structural variation described in passerine genomes, suggesting a broader role for such rearrangements in avian genome evolution. Its combination of recessive lethality, transmission bias, and extensive gene amplification provides a unique system to explore how complex chromosomal polymorphisms are maintained in natural populations. Understanding the evolutionary forces shaping this variant, whether selection on linked loci, genomic drive, or ecological factors, will require integrating karyotypic, genomic, ecological, and phenotypic data from populations, supported by long-read assemblies, transcriptomic analyses, and experimental crosses.

## 5. Conclusions

In this study, we describe an exceptionally complex structural variant of chromosome 1A in the Siberian population of the great tit. This polymorphism combines a ~55 Mb paracentric inversion on the long arm with a massive (>30 Mb) copy-number expansion on the short arm, an unusual feature for avian karyotypes. Using synaptonemal complex analysis, Hi-C, and whole-genome sequencing, we delineated the inversion breakpoints, demonstrated strong suppression of recombination across both rearranged regions, and identified multiple loci that underwent extensive amplification.

A particularly remarkable component of this amplification is a previously unassembled *FAM118A*-homologous locus, which is already multicopy in the wild type but shows an approximately twentyfold expansion in the rearranged variant. Strikingly, different *FAM118A* copies harbor varying numbers of a 630 bp tandem repeat encompassing exon 3, with the total repeat count reaching ~50,000 in the rearranged chromosome—accounting for most of the short-arm expansion.

Our analyses suggest that at least some *FAM118A* copies may remain functional, including those containing multiple exon 3 units, whereas others likely produce truncated proteins. These conclusions, however, should be treated with caution, as they are based on low-coverage Oxford Nanopore long-read data from heterozygotes and lack corresponding long-read data from wild-type homozygotes. High-quality, phased assemblies of both chromosomal variants will be essential to reconstruct the rearrangement’s architecture in detail, and functional assays are needed to determine whether the expanded *FAM118A* copies have biological significance.

Nevertheless, our results uncover previously overlooked genomic complexity on chromosome 1A, revealing multiple copies of the conserved *FAM118A* gene that may be linked to innate immune function. The nested repeat structure of the rearranged variant represents a striking and technically challenging chromosomal configuration, offering a unique opportunity to examine the mechanisms driving the origin and maintenance of large-scale structural change in avian genomes. Furthermore, the detection of an apparently identical rearrangement in European populations suggests a shared ancient origin and broad geographic distribution, raising the possibility of adaptive significance that merits targeted experimental investigation.

## Figures and Tables

**Table 1:** Summary of animal use and allocation to experimental procedures.

Experimental Procedure	Genotype	Sex	Number of Birds	Total Number of Birds per Procedure
Population karyotyping	Mixed *	Male, female	46 *	46 *
Synapsis and recombination analysis	PMA1A^S/S^	Male, female	14	19
	PMA1A^S/M^	Male, female	5	
Hi-C sequencing	PMA1A^S/S^	Female	1	2
	PMA1A^S/M^	Female	1	
Whole-Genome Sequencing (WGS)	PMA1A^S/S^	Female	2	4
	PMA1A^S/M^	Male	2	
Nanopore sequencing	PMA1A^S/M^	Female	1	1

* The population sample included individuals of all encountered genotypes. Some birds were used in multiple procedures.

**Table 2:** Frequency of PMA1A cytotypes in the Siberian population of the great tit.

	N PMA1A^M/M^	N PMA1A^S/M^	N PMA1A^S/S^	N Total	Frequency of PMA1A^S/M^	Frequency of PMA1A^M^	χ^2^ Test for HWE	*p*-Value
FEMALES	0	4	13	17	0.24 ± 0.11	0.12 ± 0.06	0.3	0.6
Males	0	4	22	26	0.15 ± 0.07	0.08 ± 0.04	0.2	0.7
Pooled	0	8	35	43	0.19 ± 0.06	0.09 ± 0.03	0.5	0.5

**Table 3:** Comparison of structural variants in great tit chromosome 1A identified in Siberian (this study) and European (da Silva et al. [40]) populations based on reference assembly Parus_major1.1 (GCF_001522545.3).

Siberian Population		European Population	
**Variant**	Coordinates, Mb	Variant	Coordinates, Mb
Inversion	3.50–56.87	Inversion	3–68
CNV1	65.86–65.90	CNV1	65.87–65.90
CNV2	67.56–67.58	CNV2	67.56–67.58
CNV3	67.64–67.66	CNV3	67.64–67.65
CNV4	63.45–63.46	CNVup1	63.44–63.46
CNV5	63.46–63.56	CNVup2	63.46–63.56
CNV6	64.818–64.824	-	-
CNV7	64.824–64.828	-	-

## Data Availability

The original data presented in this study are openly available in the Sequence Read Archive (SRA) under accession number PRJNA1376226. The final assembly Parus_major1.1_p2 and the Supplementary Files are deposited in Figshare at https://doi.org/10.6084/m9.figshare.30818327. Custom scripts are publicly available at https://github.com/genomech/TitHiC (accessed on 10 December 2025).

## Supplementary Materials

The following supporting information can be downloaded at: https://www.mdpi.com/article/10.3390/cells15010052/s1, Table S1: Primers used to generate hybridization probe for CNV8; Table S2: Coordinates, lengths, and coverage statistics of CNV regions identified in the PMA1A locus of homozygous (PMA1A^S/S^) and heterozygous (PMA1A^S/M^) birds, based on the reference PMA1A assembly Parus_major1.1 (GCF_001522545.3); Table S3. Coordinates, lengths, and coverage statistics of CNV regions identified in the PMA1A locus of homozygous (PMA1A^S/S^) and heterozygous (PMA1A^S/M^) birds, based on the updated assembly Parus_major1.1_p2; Table S4: Percent identity statistics for CNV alignments to Nanopore reads; Figure S1: K-mer (k = 80) frequencies estimated from Hi-C read pairs with one mate mapped to PMA1A and the other unmapped, in homozygous PMA1A^S/S^ versus heterozygous PMA1A^S/M^ birds; Figure S2: Metaphase plates from fibroblast cell culture of female great tit heterozygous for PMA1A^S/M^; Figure S3: Hi-C contact maps of PMA1A in the homozygote PMA1A^S/S^ (a) and the heterozygote PMA1A^S/M^ (b), built using the NCBI reference assembly Parus_major1.1 (GCF_001522545.3) at 100 kb resolution; File S1: The correspondence of coordinates between the final assembly version Parus_major1.1_p2 and NCBI reference assembly Parus_major1.1; File S2: Consensus sequence of the *FAM118A*-homologous region from the rearranged PMA1A chromosome of the great tit; File S3: Output of the miniprot alignment of the predicted FAM118A protein sequence of the blue tit (Cyanistes caeruleus; XP_023774836.1) to the great tit *FAM118A*-homologous consensus sequence.
