# Supplementary material for: An Exceptionally Complex Chromosome Rearrangement in the Great Tit (Parus major): Genetic Composition, Meiotic Behavior and Population Frequency
Source: Cells. 2025 Dec 27;15(1):52. doi: 10.3390/cells15010052 (PMC12786263; doi:10.3390/cells15010052)
Supplement: Supplementary file 1 [file cells-15-00052-s001.zip › Supplementary_Tables_Figures.pdf]

Supplementary Materials for:

An exceptionally complex chromosome rearrangement in the great tit (*Parus major)*: genetic composition, meiotic behavior and population frequency

Anna Torgasheva[1,2,3], Lyubov Malinovskaya[1,4], Miroslav Nuriddinov[1,4,5], Kira S. Zadesenets[1,4], Maria Gridina[1,4,5], Artem Nurislamov[1,4,5], Svetlana Korableva[1,4], Inna Pristyazhnyuk[1,5], Anastasiya Proskuryakova[6], Katerina V. Tishakova[1,4,6], Nikolay B. Rubtsov[1,4], Veniamin S. Fishman[1,4,5], Pavel Borodin[1,4]

[1] Institute of Cytology and Genetics, Russian Academy of Sciences, 630090 Novosibirsk, Russia;

[2] Centre for Molecular Biodiversity Research, Leibniz Institute for the Analysis of Biodiversity Change, Museum Koenig Bonn, 53113 Bonn, Germany

[3] Bonn Institute for Organismic Biology – Animal Biodiversity, University of Bonn, 53121 Bonn, Germany

[4] Laboratory of Genome Structure and Function, Novosibirsk State University, 630090 Novosibirsk, Russia

[5] Genetics and Life Sciences, Sirius University of Science and Technology, Sirius Federal Territory, 354340 Sochi, Russia

[6] Institute of Molecular and Cellular Biology, Russian Academy of Sciences, 630090 Novosibirsk, Russian Federation

*Correspondence: a.torgasheva@leibniz-lib.de

This PDF file includes:

Table S1 to S4

Figures S1 to S3

Table S1. Primers used to generate hybridization probe for CNV8

ID	Sequence (3”-5”)	Product length
CNV8_F	AGAGGCACGATGGTTTTGACT	440
CNV8_R	ACAGGAACCCACTGTCCATC	

Table S2. Coordinates, lengths, and coverage statistics of CNV regions identified in the PMA1A locus of homozygous ($PMA1A^{S/S}$) and heterozygous ($PMA1A^{S/M}$) birds, based on the reference assembly Parus_major1.1 (GCF_001522545.3).

Sample	Statistics	no CNV	CNV1	CNV2	CNV3	CNV4	CNV5	CNV6	CNV7	Total additional length, kb
	Coordinates in the PMA1A assembly*, kb	0-63,000; 68,000 - 72,000	65,863.1 - 65,901.3	67,564.1 - 67,584.7	67,633.6 - 67,657.0	63,445.6 - 63,459.7	63,460.0 - 63,557.2	64,818.3 - 64,823.7	64,824.1 - 64,827.5	
	Length of CNV, kb	-	38.2	20.6	23.4	14.1	97.2	5.4	3.3	
$PMA1A^{S/S}$ rep.1	Coverage	32	34	32	33	40	31	28	614	
	Copy number	2	2	2	2	2	2	2	38	
	Additional length, kb	-	0	0	0	0	0	0	118.8	118.8
$PMA1A^{S/S}$ rep.2	Coverage	29	31	29	27	36	29	30	688	
	Copy number	2	2	2	2	2	2	2	47	
	Additional length, kb	-	0	0	0	0	0	0	148.5	148.5
$PMA1A^{S/M}$ rep.1	Coverage	36	436	426	418	7,346	290	301	6,979	
	Copy number	2	24	24	23	408	16	17	388	
	Additional length, kb	-	840.4	453.2	491.4	5,724.6	1,360.8	81.0	1,273.8	10,225.2
$PMA1A^{S/M}$ rep.2	Coverage	32	307	296	287	5,927	223	241	5,864	
	Copy number	2	19	19	18	370	14	15	367	
	Additional length, kb	-	694.4	350.2	374.4	5,188.8	1,166.4	70.2	1,204.5	9,003.1

* NCBI reference assembly Parus_major1.1 (GCF_001522545.3)

Table S3. Coordinates, lengths, and coverage statistics of CNV regions identified in the PMA1A locus of homozygous (PMA1A$^{S/S}$) and heterozygous (PMA1A$^{S/M}$) birds, based on the updated assembly Parus_major1.1_p2.

Sample	Statistics	no CNV	CNV1	CNV2	CNV3	CNV4	CNV5	CNV6	CNV7a*	CNV8	Total additional length, kb
	Coordinates in the PMA1A assembly**, kb	0-64,500; 70,500 - 71,500	67,962.4 - 68,000.7	69,663.4 - 69,684.0	69.732.9 - 69,756.3	64,757.3 - 64,771.1	64,771.5 - 64,868.7	64,736.3 - 64,741.7	64,742.1 - 64,748.9; 64,749.5 - 64,757.3	64,748.9 - 64,749.5	
	Length of CNV, kb	-	38.2	20.6	23.4	13.7	97.2	5.4	14.6	0.6	
PMA1A$^{S/S}$ rep.1	Coverage	32	33	31	32	40	31	28	668	102,197	
	Copy number	2	2	2	2	2	2	2	42	6,387	
	Additional length, kb	-	0	0	0	0	0	0	582.2	4,016.2	4,598.4
PMA1A$^{S/S}$ rep.2	Coverage	29	31	29	27	36	28	30	733	119,465	
	Copy number	2	2	2	2	2	2	2	51	8,239	
	Additional length, kb	-	0	0	0	0	0	0	713.2	5,181.1	5,894.3
PMA1A$^{S/M}$ rep.1	Coverage	36	436	426	418	7,334	289	300	7,547	805,567	
	Copy number	2	24	24	23	407	16	17	419	44,754	
	Additional length, kb	-	840.7	453.2	491.0	5,558.6	1,360.9	815.5	6,069.4	28,149.0	43,004.3
PMA1A$^{S/M}$ rep.2	Coverage	32	307	296	287	5,926	222	240	6,239	760,998	
	Copy number	2	19	19	18	370	14	15	390	47,562	
	Additional length, kb	-	649.6	350.2	374.1	5,050.8	1,166.5	70.6	5,647.3	29,915.2	43,224.3

* CNV7a consists of two fragments of the *FAM118A*-homologous consensus sequence, separated by CNV8 for computational purposes; its proximal fragment fully contains CNV7.

** Updated reference assembly Parus_major1.1_p2.

Table S4. Percent identity statistics for CNV alignments to Nanopore reads

CNV ID	CNV1	CNV2	CNV3	CNV4	CNV5	CNV6	CNV7	CNV8
CNV length, bp	38,211	20,599	23,380	13,912	97,208	5,429	3,331	630
Number of hits	608	611	598	610	728	121	554	15,187
Median block length, bp	1,856	485	786	13,682	3,536	1,590	3,274	628
Mean block length, bp	3,958	2,396	3,053	11,937	69,182	2,511	3,213	615
Median % identity	95.8	89.5	90.7	96.7	96.3	93.8	95.4	98.4
Mean % identity	93.8	89.7	91.6	96.0	94.4	90.1	95.2	98.0
Length-weighted mean % identity*	94.9	93.7	94.1	96.1	95.6	93.7	95.2	98.0
25th percentile % identity	92.4	83.5	89.6	95.6	93.6	82.2	94.8	97.6
75th percentile % identity	97.0	96.1	95.7	97.1	97.4	95.6	95.8	99.0

* mean percent identity weighted by alignment length

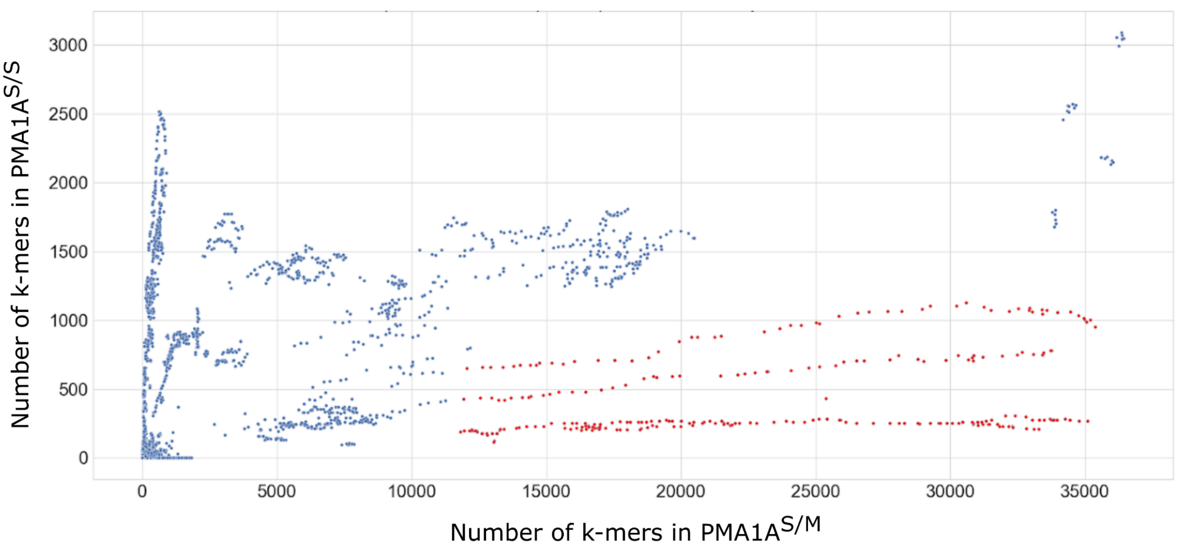

Figure S1. K-mer (k = 80) frequencies estimated from Hi-C read pairs with one mate mapped to PMA1A and the other unmapped, in homozygous (PMA1A$^{S/S}$) versus heterozygous (PMA1A$^{S/M}$) birds. Each dot represents a single k-mer. Red dots represent k-mers significantly enriched in the heterozygote selected for subsequent analysis.

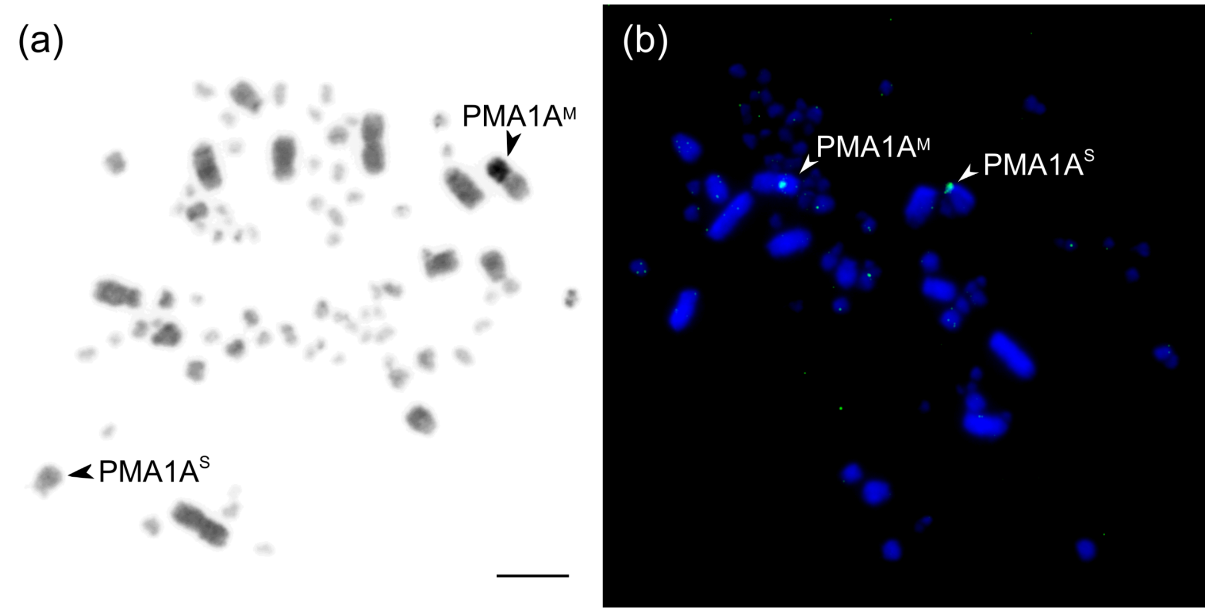

Figure S2. Metaphase plates from fibroblast cell culture of heterozygous (PMA1A$^{S/M}$) female great tit. **(a)** C-banding followed by DAPI staining (inverted). **(b)** FISH with the BAC clone CH261-36B5 specific to GGA1p (green) followed by DAPI staining. Scale bar - 5 μm.

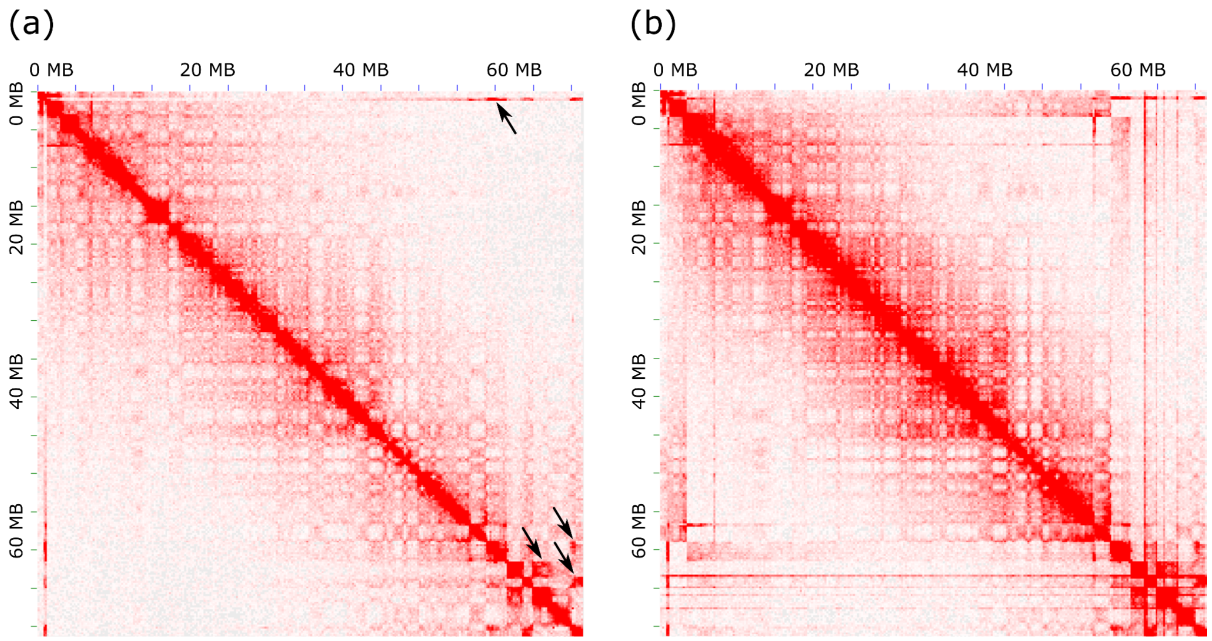

Figure S3. Hi-C contact maps of PMA1A in the homozygote PMA1A$^{S/S}$ (**a**) and the heterozygote PMA1A$^{S/M}$ (**b**), built using the NCBI reference assembly Parus_major1.1 (GCF_001522545.3) at 100-kb resolution. Arrows indicate regions with Hi-C contact patterns suggestive of population-specific rearrangements or misassemblies in the reference PMA1A.
